# Efficacy of photodynamic therapy on candida colonization and clinical symptoms in denture stomatitis: a systematic review and meta-analysis

**DOI:** 10.1186/s12903-023-03789-z

**Published:** 2024-01-16

**Authors:** Sadeq A. Al-Maweri, Mohammed Nasser Alhajj, Lamyia Anweigi, Sajna Ashraf, Esam Halboub, Nosizana Mohd Salleh, Rawan H. Alanazi, Mohammad Zakaria Nassani, Mohammed Noushad, Jumma O. Al Khabuli, Anas Shamala

**Affiliations:** 1 College of Dental Medicine, QU Health, Qatar University, Doha, Qatar; 2 Department of Restorative Dentistry, Faculty of Dentistry, Federal Territory of Kuala Lumpur, Universiti Malaya, Kuala Lumpur, Malaysia; 3 Department of Prosthodontics, Faculty of Dentistry, Thamar University, Dhamar, Yemen; 4 Department of Oral Medicine and Diagnostic Sciences, Vision Colleges, Riyadh, Kingdom of Saudi Arabia; 5 Department of Maxillofacial Surgery and Diagnostic Sciences, College of Dentistry, Jazan University, Jazan, Saudi Arabia; 6 Department of Department of Oral and Maxillofacial Surgery, Vision College of Dentistry and Nursing, Riyadh, Saudi Arabia; 7 Department of Restorative and Prosthetic Dental Sciences, College of Dentistry, Dar Al Uloom University, Riyadh, Saudi Arabia; 8 Department of Preventive and Biomedical Science, College of Dentistry, University of Science & Technology, Sanaa, Yemen

**Keywords:** Photodynamic therapy, Denture stomatitis, Management, Systematic review

## Abstract

**Background:**

Photodynamic therapy (PDT) has been recently proposed as a promising alternative therapy for Denture Stomatitis (DS). The present systematic review and meta-analysis investigated the current available evidence regarding the efficacy of PDT in the management of DS.

**Materials and methods:**

PubMed, Scopus, Web of Science, Google Scholar, and ProQuest were searched up to June 7, 2023. All relevant clinical trials were included. RevMan software was used for the statistical analyses.

**Results:**

Elven randomized clinical trials (460 DS patients) were included. Eight studies assessed the efficacy of PDT vs. topical antifungal therapy, while three studies assessed the adjunctive use of PDT (PDT + antifungal therapy) vs. topical antifungal therapy alone. The results revealed comparable efficacy of PDT and conventional antifungal therapy on candida colonization at 15 days (MD: 0.95, 95% CI: -0.28, 2.19, *p* = 0.13) and at the end of follow-up (MD: -0.17, 95% CI: -1.33, 0.98, *p* = 0.77). The pooled two studies revealed relatively better efficacy of adjunctive use of PDT with antifungal therapy on candida colonization compared to antifungal therapy alone at 15 days (MD: -6.67, 95% CI: -15.15, 1.82, *p* = 0.12), and at the end of follow-up (MD: -7.14, 95% CI: -19.78, 5.50, *p* = 0.27). Additionally, the results revealed comparable efficacy of PDT and topical antifungal therapy on the clinical outcomes.

**Conclusions:**

PDT might be considered a viable option for DS either as an adjunct or as an alternative to the topical antifungal medications. Further studies with adequate sample sizes and standardized PDT parameters are warranted.

**Supplementary Information:**

The online version contains supplementary material available at 10.1186/s12903-023-03789-z.

## Introduction

Denture stomatitis (DS) is a very common oral inflammatory condition affecting 15% to 70% of removable denture wearers [1–4]. A considerable portion of DS cases are asymptomatic and discovered incidentally during dental examination as an erythema and/or edema of the oral mucosa covered by the denture [4]. However, some DS patients may complain of pain, itching and/or burning [4]. Although DS is a relatively common disorder, its exact etiology has not yet been entirely understood [4, 5]. By and large, there is an agreement that DS is a multifactorial disease [4]. *Candida Albicans* has been found to be strongly associated with, and even reported to be implicated in pathogenesis of DS [3, 4, 6, 7]. Dentures are usually fabricated from polymethyl methacrylate resin with its inherited porosity disadvantage [8]. The fungal species, mainly *Candida Albicans*, colonize the porous surface of the acrylic resin, causing oral mucosal inflammation [8]. Other systemic and local predisposing factors include, but not limited to, trauma from ill-fitting denture, poor oral and/or denture hygiene, smoking, decreased salivary flow, medications, increased age of denture, continuous wearing of the denture, and systemic diseases, like diabetes mellitus [5–7, 9, 10]. These factors appear to increase the ability of opportunistic fungal pathogens, mainly *Candida albicans*, to colonize both the denture and oral mucosal surfaces causing inflammation [3, 4, 11].

Beside adjusting and managing the aforementioned predisposing factors, topical and systemic antifungal medications are still the mainstay treatment of DS [12]. However, these medications are not always effective in eradicating the fungal colonies from the dentures, and may be associated with a high risk of recurrence after antifungal therapies [12–15]. Another significant limitation of antifungal therapies is that fungal species may develop resistance against these medications especially in patients with long-term use [16]. Moreover, the long-term use of these medications, especially systemic antifungals, is usually associated with various side effects including the risk of drug interactions, a matter that limits their use. The above argument justifies seeking for alternative novel therapies for DS that are safe, effective, and well-tolerated, without the disadvantages of conventional therapies.

Photodynamic therapy (PDT) has been proposed as a novel, promising treatment modality for several oral mucosal conditions, including DS [17–19]. PDT is a two-stage treatment involving application of a light-sensitive chemical substance –called a photosensitizer- followed by application of a visible light radiation [14, 20]. In the presence of oxygen, the interaction between the photosensitizer and radiation results in production of singlet oxygen and other oxygen reactive species causing cell damage and death of the microorganism, with minimal damaging effects on the host cells [14]. Additionally, PDT has been suggested to have anti-inflammatory and immunomodulatory properties, a matter that explains its therapeutic effects [17, 21].

In context of DS, a number of clinical trials have evaluated the efficacy of PDT, and reported promising results [14, 15, 22–26]. A 2018-study by de Senna et al. [15] compared the efficacy of PDT with topical antifungal therapy in DS, and found equivalent efficacy in reducing candida count and clinical signs of DS. Two more recent clinical studies among DS patients in Saudi Arabia also replicated these results [23, 25]. On the other hand, one study by Alves et al., 2020 found PDT to be inferior to topical nystatin in reducing candida colonization in DS patients [14]. A recent 202- study by Al-Aaali et al. [27] investigated the efficacy of PDT on fungal growth and oral health related quality of life in DS patients. The results revealed superiority of miconazole gel over PDT, but a combination therapy (PDT + Miconazole) showed significantly better results than Miconazol [27]. In this context, a few systematic reviews attempted to summarize the available evidence regarding the efficacy of PDT in the management of DS, and reported conflicting results [28–30]. It is worth mentioning, however, that the aforementioned reviews included only very limited number of studies (3–5 studies) and failed to include all potentially eligible studies, and thus the results might be inconclusive. Additionally, more recent relevant clinical trials on the efficacy of PDT in DS have been published over the past two years [22, 27, 31], again with interesting results.

In light of the fact that the above mentioned limitations of the previous systematic reviews [14, 22, 24, 25] and the lack of a comprehensive systematic review addressing the effect of PDT in comparison to the topical and/or systemic antifungal in treatment of DS, the present systematic review and meta-analysis sought to analyze and update the current evidence in this regard.

## Materials and methods

### Study protocol and focused question

The protocol of the present systematic review was registered in PROSPERO (registration # CRD42021286140). The focused question was: “Is PDT efficacious in the management of DS?” The present systematic review and meta-analysis followed the Preferred Reporting Items for Systematic Review and Meta-analyses (PRISMA) guidelines and PICOS (Participants, Intervention, Comparison, Outcomes, and Study Design) principles [32].

### Eligibility criteria

The PICOS eligibility criteria of the present systematic review were: 1) Participants (P): subjects with DS; 2) Intervention (I): PDT alone or in combination with antifungal therapy; 3) Comparator (C): Topical or systemic antifungal therapies; 4) Outcomes (O): Clinical (pain, redness) and /or microbial (Candida colony counts) improvement; and 5) Study design (S): Randomized controlled clinical trials (RCT). Retrospective and prospective observational studies, case series, case reports, animal studies, review papers, editorials, letters to the editor, commentaries, conference proceeding, and monographs were excluded.

### Search strategy and information sources

A comprehensive search of multiple online databases/search engines (PubMed, Scopus, Web of Science, and Google Scholar) was conducted on June 7, 2022 for all potential studies published between January 2000 and June 2022, with no language or time restrictions. The search was updated on June, 8th, 2023. The grey literature was searched through “ProQuest”. We used a combination of the following MeSH (medical subject headings) and free keywords: ((“denture stomatitis” OR “oral candidiasis”) AND (“Photodynamic therapy” OR “photochemotherapy”)). A detailed search strategy is presented in Supplementary Table 1.

### Screening and selection process

The retrieved studies were exported to Endnote program, and duplicates were eliminated. Two investigators (SAA & RA) screened the titles and abstracts of the retrieved articles independently, and the irrelevant studies were removed. The full-text of the potentially eligible studies were obtained and thoroughly scrutinized independently by the two investigators for inclusion. The online search was supplemented with a manual search in the reference lists of the included studies.

### Data extraction

Relevant data were extracted and tabulated by two investigators independently using special forms included the following: author, year, country of publication, participants (sample size, mean age, and gender), comparison group, type of DS, evaluation methods, outcome measures, follow-up in days, type of photosensitizers, number and duration of PDT sessions, and the main outcomes.

### Quality assessment

The methodological quality of the included studies was assessed independently by two reviewers (SA, NA) using the Cochrane risk-of-bias assessment tool [33], and disagreements, if present, were resolved by discussion and/or by consulting a third reviewer. Six domains were evaluated: sequence generation, allocation concealment, blinding of participants and personnel, blinding of outcome assessment, incomplete outcome data, and selective outcome reporting. Accordingly, the quality of each study was graded as either: low, all items were of low risk; high, at least one item with high risk of bias; or unclear, at least one item was evaluated to be of unclear risk but no item of high risk [33].

### Statistical analysis

Statistical analysis was performed using Review Manager (RevMan) Version 5.3. Copenhagen: The Nordic Cochrane Centre, The Cochrane Collaboration, 2014. The meta-analyses were conducted by calculating the mean difference between the groups along with 95% confidence intervals (CIs) for continuous outcomes, and by calculating the odds ratios (OR) along with 95% CIs for dichotomous outcomes. Heterogeneity between studies was evaluated using Chi-square test and the I^2^ statistics [34]. Fixed-effects model was used for low/moderate heterogeneity (I^2^ ≤ 50%), while random effect model was applied for significant heterogeneity (I^2^ > 50%). The potential publication bias was assessed using the funnel plots [35]. Due to the limited number of the included studies, no sensitivity tests or subgroup analysis were conducted.

## Results

### Study selection

**Fig. 1:**
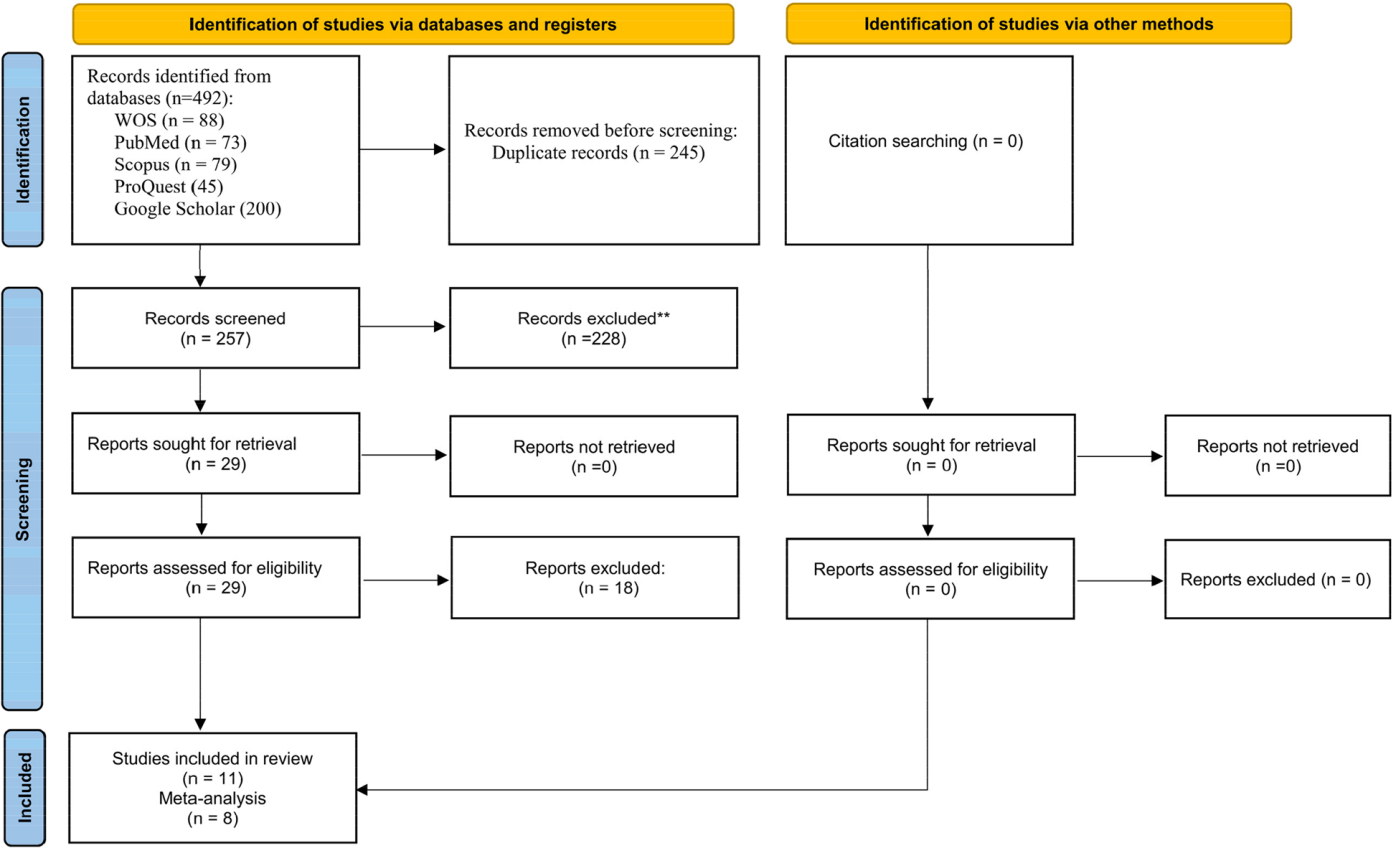
Flow diagram of the search strategy

Figure 1 present the results of the search strategy. A total of 492 articles were identified from the online searches (PubMed 73, Web of Science 88, Scopus 79, Google Scholar 200, ProQuest 45). Of these, 235 articles were duplicates and thus were excluded. The titles and abstracts of the remaining 257 were screened, and 228 were found irrelevant (reviews, in-vitro studies, case reports, case series, conference proceedings, or irrelevant to the focused question), and hence they were excluded. The full-text of the 29 potentially eligible studies were obtained, and thoroughly scrutinized for inclusion. Of these, 18 articles were excluded for various reasons (reviews, irrelevant outcome of interest, See Supplementary Table 2). Eventually, 11 studies were included in the systematic review [14, 15, 22–27, 31, 36, 37], eight of which were eligible for meta-analysis.

### General characteristics

General characteristics of the included studies are summarized in Table 1. A total of 11 randomized controlled studies comprising 450 DS patients aged between 40 and 80 years were included in the present systematic review [14, 15, 22–27, 31, 36, 37]. The studies were published between 2011 and 2023. The number of subjects in each included study ranged between 22 and 65. Five studies [14, 15, 22, 26, 37] were conducted in Brazil, four [23, 25, 27, 31] in Saudi Arabia, one [24] in India, and one in Iran [36]. Only eight studies [14, 15, 23, 25–27, 31] reported gender of the participants, where the majority were females. The follow-up period ranged from 30 to 90 days.

**Table 1:** General characteristics of the included RCT studies

**Author Year**	**country**	**Intervention (PDT) Sample size M/F**	**Control group**	**Type of DS**	**Evaluation methods**	**Outcome measures (Follow-up)**	**Main outcomes**
Labban et al. 2021 [25]	Saudi Arabia	**G1:** RBM- PDT *N* = 15 M/F: 3/12 **G2 II**: CM- PDT *N* = 15 M/F: 5/10	Nystatin topical oral suspension 100,000UL/mL 4 times/day/2 weeks *N* = 15 M/F = 4/11	Type I,II,III	Microbial, Clinical	Candida colony counts, Clinical resolution **Follow up:** 6 weeks, 12 weeks	PDT was as effective as nystatin
Alonso et al. 2021 [22]	Brazil	PDT *N* = NM M/F = NM	Nystatin topical oral suspension 100,000UI/mL 4 times/day/2 weeks *N* = NM	NM	Microbial	Candida colony counts, Prevalence of candida species **Follow up:**45 days	PDT was as effective as nystatin
Jaisinghani et al. 2021	India	PDT *N* = 20 M/F = NM	Clotrimazole paint 3 times/day/30 days *N* = 20 M/F = NM	Type II	Clinical	Size of the lesions **Follow up:** 15 days, 30 days	PDT showed significantly better results than clotrimazole mouth paint
Alves et al. 2020 [14]	Brazil	PDT *N* = 30 M/F:11/19	Nystatin topical oral suspension 100,000UL/mL 4 times/day/2 weeks *N* = 35 M/F:9/24	Type I,II,III	Microbial, Clinical	Candida colony counts, Prevalence of candida & Resolution of lesions **Follow up:** 15,30,45 days	Nystatin showed significantly better results in reducing candida species, and comparable results in clinical improvement
Alrabiah et al. 2019 [23]	Saudi Arabia	PDT *N* = 18 M/F: NM Age: NM	Nystatin topical oral suspension 100,000 IU 4 times/day/2 weeks *N* = 18, M/F: NM	NM	Microbial	Candida colony counts **Follow up:** 15,30,60	PDT was as effective as nystatin
de Senna et al. 2018 [15]	Brazil	PDT *N* = 18 M/F: 1/17	Oral miconazole gel 2% 3 times/day/30 days *N* = 18 M/F: 1/17	NM	Clinical, Microbial	Counting, and identification of species, degree of erythema **Follow up:**15, 30 days	PDT was as effective as miconazole in reducing candida as well as clinical signs of denture stomatitis
Mima et al., 2012 [26]	Brazil	PDT *N* = 20 M/F: 7/13	Nystatin topical oral suspension 100,000 IU 4 times/day/2 weeks *N* = 20 M/F: 5/15	Type I, II, III	Clinical, microbial	Candida colony counts, Prevalence of Candida spp. palatal erythema, **Follow up:** 15,30,60,90 days	PDT was as effective as nystatin
Lopes 2011 [37]	Brazil	PDT *N* = 12 NA	Nystatin topical oral suspension 100,000 IU 6 times/ day for 2 weeks *N* = 10	NA	Microbial	Candida colony counts, Follow-up: 30 days	PDT was superior to nystatin at 7 and 14 days, but comparable results at 30 days
Al-Aali et al. 2023 [27]	Saudi Arabia	G1: PDT *N* = 20 7/13 G2: PDT + Miconazole gel 2% *N* = 20 8/12	Miconazole gel 2% 4 times/day *N* = 20 5/15	Type I, II, III	Microbial, Quality of life	Candida colony counts Follow-up: 60 days	Miconazole gel was more efficacious in day 144, but comparable with PDT at end of the follow-up. Combination of PDT and miconazole and significantly better results
Afroozi et al. 2019 [36]	Iran	PDT + nystatin *N* = 28 6/20 Age: 67.6	Nystatin topical oral suspension 100,000 IU 3 times/ day for 2 weeks *N* = 28 7/19 Age: 67.6	Type I, II, III	Clinical, microbial	Candida colony counts, erythema, recurrence Follow-up: 60 days	PDT + nystatin group showed significantly better results than nystatin alone
Al-Ghamdi et al. 2023 [31]	Saudi Arabia	PDT + Miconazol gel 2% *N* = 25 10/15 Age: 55.2	Miconazol gel 2% 4 times/day for 15 days *N* = 25 9/16 Age: 56.7 years	NM	Clinical Microbial Inflammat-ory cytokines	Candida colony counts,ELIZA erythema	PDT + miconazole groups was significantly more efficacious

*RCT* Randomized controlled trials, *PDT* Photodynamic therapy, *DS* Denture stomatitis, *M* Male, *F* Female, *NM* Nnot mentioned, *RBM- PDT* Rose Bengal-mediated PDT, *CM* Curcumin mediated

With respect to the intervention groups, eight studies assessed the efficacy of PDT in comparison to topical antifungal therapy; two studies compared the efficacy of adjunctive PDT (PDT + topical antifungal) in comparison to antifungal therapy alone; and one study compared three groups: G1, PDT alone; G2, a combination therapy (PDT + antifungal therapy); and G3, Topical antifungal. All included studies used topical antifungal therapy as a comparator group. Of these, seven studies [14, 22, 23, 25, 26, 36, 37] used topical Nystatin oral suspension 100,000UL/mL (four times/day for 15 days), three studies [15, 27, 31] used topical miconazole 2% gel, and one study [24] used Clotrimazole paint (four times/day for 15 days).

### Photosensitizers and laser related parameters

The included studies varied greatly with respect to the number of PDT sessions, treatment duration, photosensitizers, and laser-related parameters. With respect to photosensitizers**,** three studies used methylene blue 5% [15, 23, 24], two studies used photodithazine [14, 22], one study used photogem (a haematoporphyrin derivative) [26], one study used methylene chloride [37], and one study used curcumin [25]. In all studies, the photosensitizer was applied topically. The pre-irradiation time ranged from 10–30 min. Number of PDT sessions ranged from 2–6 sessions (Table 3). Six studies [15, 23, 24, 27, 36, 37] used diode laser whereas five studies [14, 22, 25, 26, 31] used light emitting diode (LED). The wavelengths and the power density of laser ranged from 455 to 940 nm and 40 to 240 mW cm^2^, respectively (Table 2).

**Table 2:** Characteristics of photosensitizers and laser parameters used in the included studies

Authors	Type of photosensitizer And %	Rout of administration	Light source	Pre-irradiation Time (in minutes)	Treatment sessions and frequency	Laser Wavelength (in nm)	Power density (mW/cm^2^)
Labban et al., 2021 [25]	G1: Rose Bengal 5 μg/ml G2: Curcumin 5 μg/ mL	Topical spray 5ml on palate and denture	LED (Royal blue)	30 min	6 sessions; (thrice/week)	455 nm	Denture: 24 Palate:102
Alonso et al., 2021 [22]	Photodithazine 200 mg/L	Topical gel	LED	20 min	6 sessions; (thrice/week)	660 nm	Denture: 50 Palate: 240
Jaisinghani et al., 2021	methylene blue (aqueous stain solution)	Topical	Diode laser	NA	4 sessions; twice/week	940 nm	200
Alves et al., 2020 [14]	5 mL of Photodithazine at 200 mg/L	hydrogel	LED (red)	20 min	6 sessions; (thrice/week)	660 nm	Denture: 50 Palate: 240
Alrabiah et al., 2019 [23]	methylene blue 450 μg/mL	Topical spray 5ml on palate and denture	GaA1As diode laser	10 min	4 sessions; 2/week	660 nm	100
De Senna et al. 2018 (Brazil) [15]	methylene blue 450 μg/mL	applied using a cotton swab	GaA1As diode laser	10 min	4 sessions; 2/week	660 nm	100
Mima et al., 2012 [26]	Photogem (haematoporphyrin Derivative) 500 mg/L	Topical spray 5ml on palate and denture	LED (Royal blue)	30 min	6 sessions; (thrice/week)	455 nm	Denture: 24 Palate:102
Lopes, 2011 [37]	0.005% methylene chloride	Topical	Diode laser	NA	2 sessions One/week	660nm	40
Al-Aali et al. 2023 [27]	Methylene blue 0.005%	Topical	Diode laser	5 min	1 session	660nm	100
Afroozi et al. 2019 [36]	indocyanine green-mediated	Topical	Diode laser	4	2 sessions	810 nm	NM
Al-Ghamdi et al. 2023 [31]	curcumin-mediated 0.8 ug/mL	Topical	LEDs	20 min	16 sessions (2 per week)	440–460	102

*GaA1As* Gallium-aluminum-arsenium, *LED* Light emitting diode

### Outcome measures

Eight studies [14, 15, 23, 25–27, 31, 36] ascertained both clinical (i.e., burning sensation and/or size of the redness) and mycological (candida colony count and/or prevalence of candida species) outcomes, while two studies [22, 37] reported on the mycological outcomes only, and one study [24] reported on clinical outcomes only.

### Main outcomes

The included results showed variable results with most of the included studies reported good efficacy of PDT in reducing the candida colony count and resolution of signs and symptoms of DS. Five studies [15, 22, 23, 25, 26] reported comparable results between PDT and the topical antifungal therapy, one study [24] found better results in favor of PDT compared to clotrimazole mouth paint in reducing the clinical signs of DS, and one study [14] reported inferior efficacy of PDT in comparison to topical nystatin in reducing the candida count, but comparable results with respect to reducing the clinical signs. One study [37] showed that PDT was superior to nystatin in reducing the candida count on days 7 and 14, while the results on day 30 of treatment were comparable.

All three studies [27, 31, 36] that assessed the efficacy of combination therapy (PDT + topical antifungal) in comparison to topical antifungal alone reported significantly lower candida colony count and better clinical improvement in favor of the combination therapy.

## Meta-analysis results

### Mycological effect of PDT vs. topical antifungal therapy

**Fig. 2:**
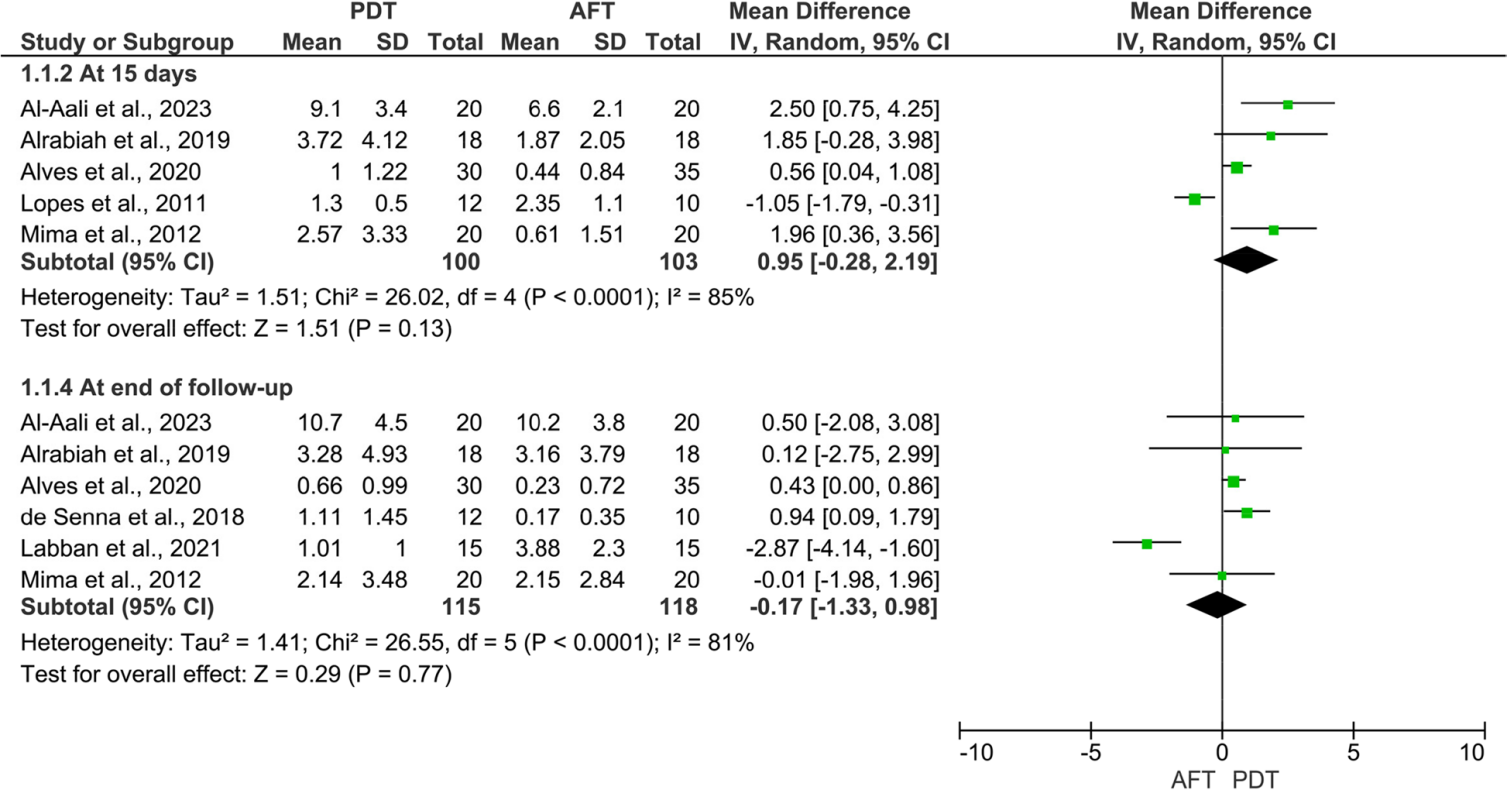
Meta-analysis of candida colony count of the palate (PDT vs Antifungal) PDT: photodynamic therapy; AFT: antifungal therapy

The results of the pooled studies revealed slightly insignificant better efficacy of topical antifungal therapy in reducing candida colonization (candida colony count) of the palatal mucosa on day 15 (I^2^ = 85%; MD = 0.0.95, 95% CI: -0.28, 2.19, *p* = 0.13), and comparable results at the end of follow-up (I^2^ = 88%; MD = -0.17, 95% CI: -1.33, 0.98, *p* = 0.77) (Fig. 2).

### *Mycological effect of adjunctive PDT (PDT* + *Antifungal therapy) vs. topical antifungal therapy:*

**Fig. 3:**
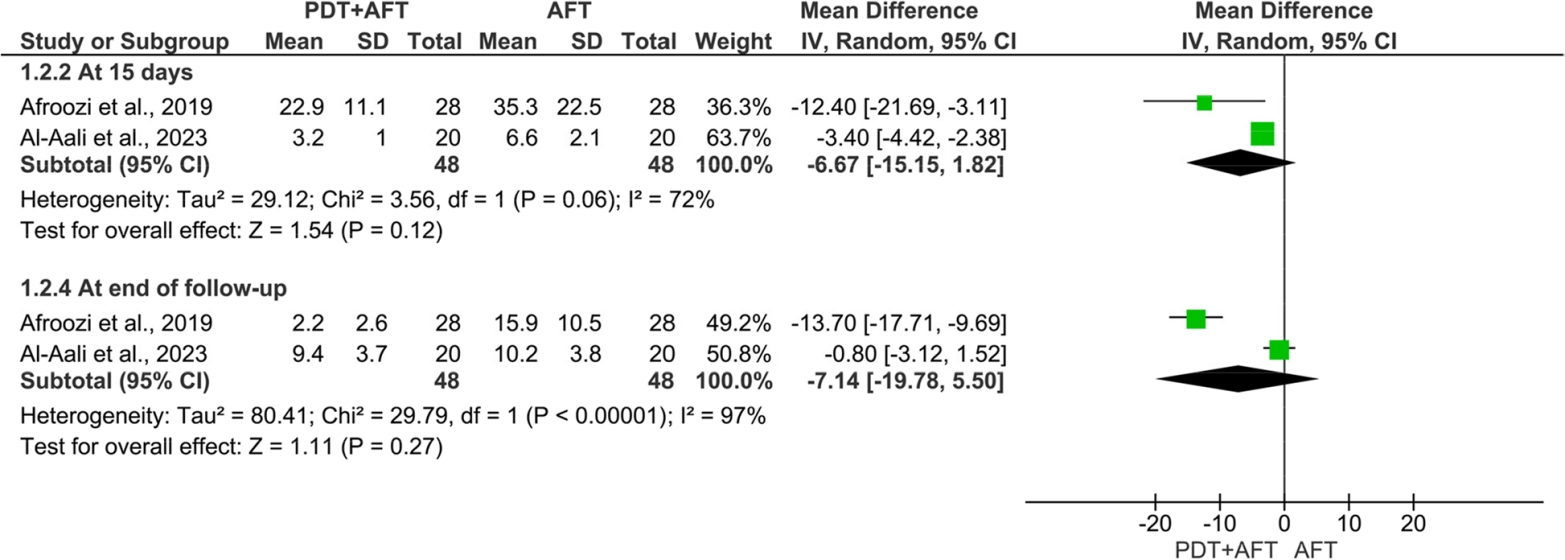
Meta-analysis of candida colony count of the palate (PDT + Antifungal vs Antifungal)

The pooled two studies revealed relative better efficacy of PDT + antifungal in reducing candida colony count compared to antifungal therapy alone on day 15 (I^2^ = 72%; MD = -6.67, 95% CI: -15.15, 1.82, *p* = 0.12), and at the end of follow-up (I^2^ = 97%; MD = -7.14, 95% CI: -19.78, 5.50, *p* = 0.27), but with no statistical differences (Fig. 3).

### Clinical efficacy

**Fig. 4:**
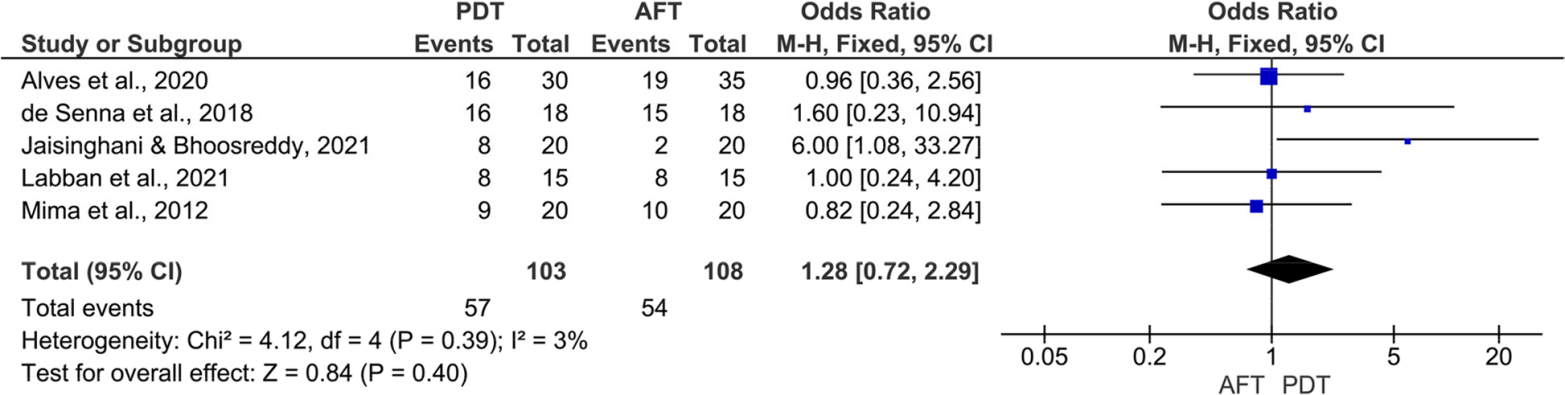
Meta-analysis of clinical efficacy

The results of pooled five studies revealed comparable efficacy of PDT and topical antifungal therapy in improvement of the clinical signs (I^2^ = 3%; OR = 1.28, 95% CI: 0.72, 2.29, *P* = 0.40) (Fig. 4).

### Publication bias

**Fig. 5:**
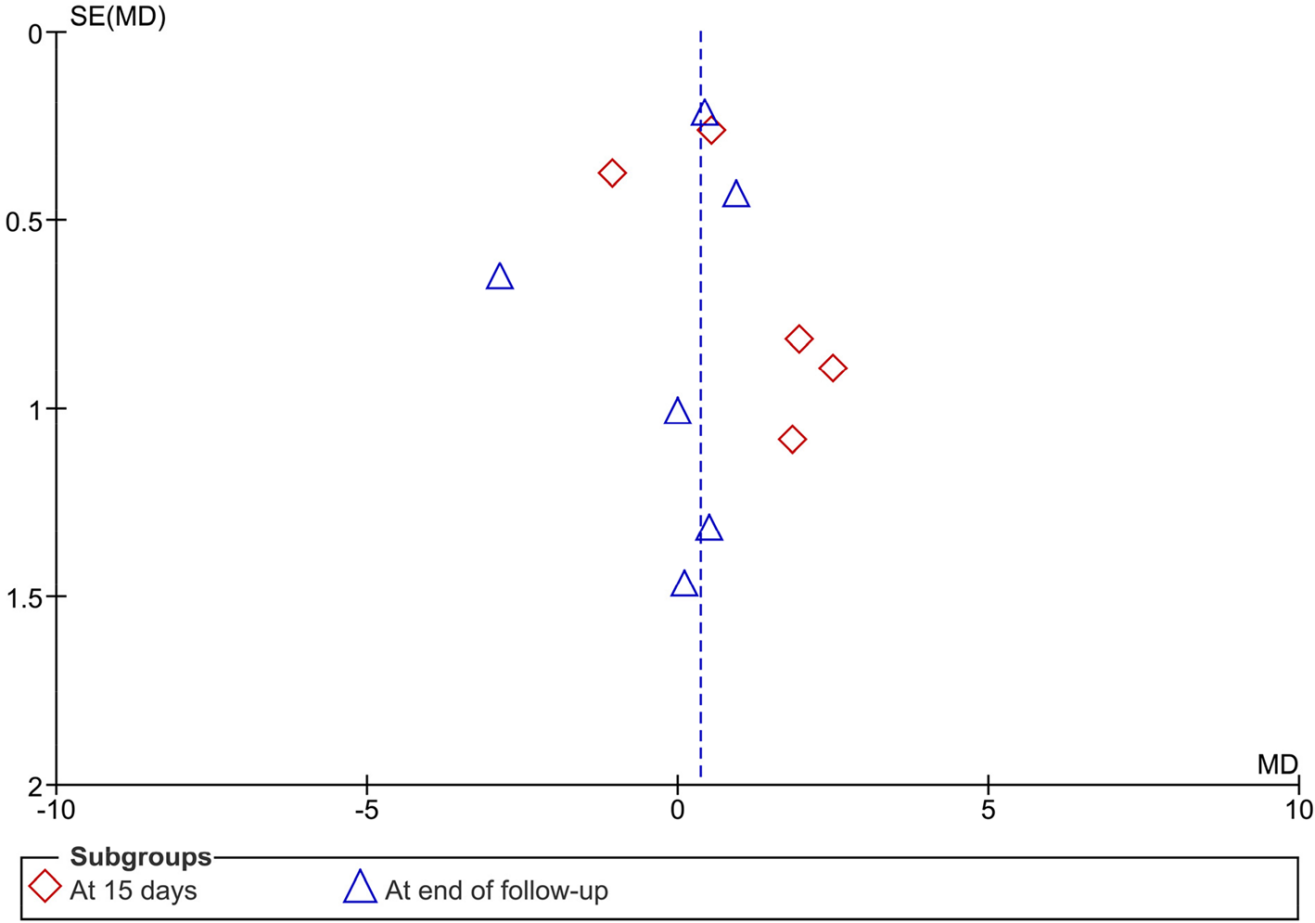
Publication bias diagram of candida colony count of the palate (PDT vs Antifungal)

**Fig. 6:**
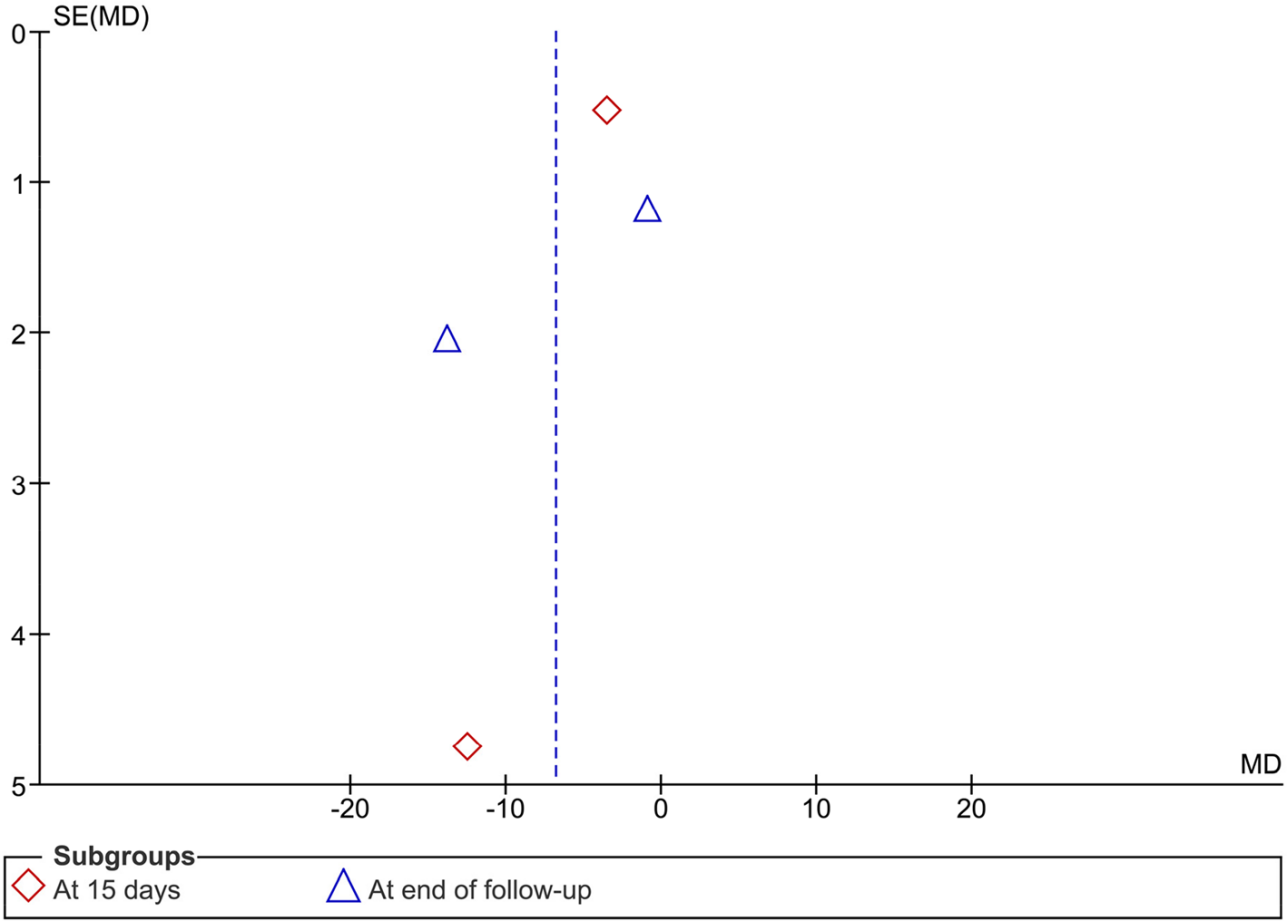
Publication bias diagram of candida colony count of the palate (PDT + Antifungal vs. Antifungal)

**Fig. 7:**
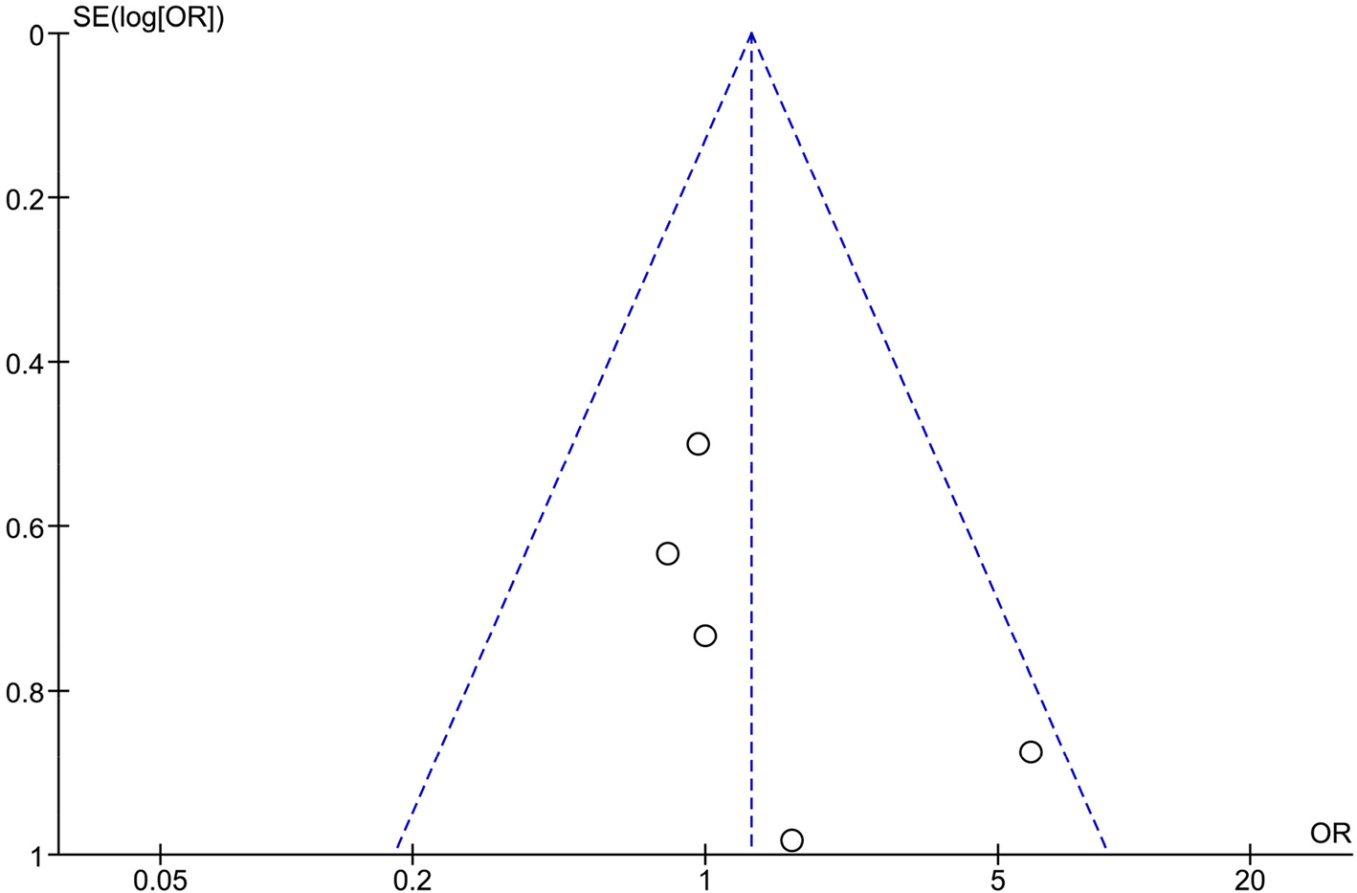
Publication bias diagram of clinical efficacy

The funnel plots showed no any sign of publication bias (Figs. 5, 6 and 7).

### Quality of the included studies

Five studies were at low risk of bias [14, 23, 25, 27, 36], three studies were at high risk of bias [15, 22, 37], and three were of unclear risk of bias [24, 26, 31]. The most frequent methodological shortcomings in these studies were unreported methods of randomization, and inadequate or lack of masking (Table 3).

**Table 3:** Risk of bias assessment results of the included studies

Study	Random Sequence generation	Allocation concealment	Blinding of participants and personnel	Blinding of outcome assessment	Incomplete outcome data	Selective reporting	Overall
Labban et al [25]	Low	Low	Low	Low	Low	Low	Low
Alonso et al	High	High	High	High	Low	Low	High
Jaisinghani et al	Low	Unclear	Low	Low	Low	Low	Unclear
Alves et al	Low	Low	Low	Low	Low	Low	Low
Alrabiah et al	Low	Low	Low	Low	Low	Low	Low
De Senna et al	High	High	High	High	Low	Low	High
Mima et al	Low	Unclear	Unclear	Unclear	Low	Low	Unclear
Lopes	Low	Unclear	High	Unclear	Low	Low	High
Al-Aali et al	Low	Low	Low	Low	Low	Low	Low
Afroozi et al. 2019 [36]	Low	Low	Low	Low	Low	Low	Low
Al-Ghamdi et al. 2023 [31]	Low	Low	Unclear	Low	Low	Low	Unclear

## Discussion

Recently, PDT has been proposed as a novel and promising therapeutic modality for various oral inflammatory diseases, including DS [17–19, 30, 38]. The present systematic review aimed to answer the focused question: “Is PDT efficacious in the management of DS as compared to the topical antifungal medications?”. The qualitative analysis of the included studies answered explicitly that PDT is as efficacious as the topical antifungal therapies, and that adjunctive PDT therapy is more efficacious than antifungal alone in the management of DS, although the statistical significant is at borderline. More specifically, the pooled results of seven studies revealed nearly equivalent efficacy of PDT and topical antifungal therapies in reducing the candida colony count and improvement of clinical signs of DS. Moreovere, the pooled two studies found better efficacy of adjunctive use of PDT (PDT + antifungal therapy) in the management of DS than antifungal alone. Nevertheless, despite these promising results, the findings of the present systematic review should be interpreted with caution given the substantial heterogeneity among the included studies and low quality in some of the included studies, as discussed in the following sections.

One of the primary outcome measures assessed in the present systematic review was the mycological efficacy of PDT. The findings revealed that PDT was very efficacious in reducing the candida colonization count from the palatal mucosa, which was equivalent to or even better than topical antifungal medications. The antimicrobial properties of PDT can be ascribed to the synergistic interaction between the photosensitizer and the radiation that results in production of singlet oxygen and other oxygen reactive species that cause cell damage and death of the microorganism [14, 20]. The findings of the present systematic review support previous systematic reviews and meta-analyses that reported strong antimicrobial efficacy of PDT, with no reported side effects [19, 28, 38, 39]. However, the present results are different from a recent meta-analysis of three studies on DS subjects, which found inferior outcomes with PDT as compared to nystatin [30]. It should be noted, however, that the latter meta-analysis included only three studies, while in our review eight studies were pooled, and this may explain the differences in the results.

Another key outcome assessed in the present systematic review was the clinical efficacy (i.e., reducing clinical signs and symptoms associated with DS) of PDT. Overall, the included studies revealed a good efficacy of PDT in reducing the size of the lesions and ameliorating the symptoms, a finding which is consistent with the previous literature. In addition to its antimicrobial action, PDT have been shown to have potent anti-inflammatory, immunomodulatory effects as well as healing promoting properties through biomodulation in irradiated tissues [21, 40]; this together may explain the therapeutic effects of PDT in alleviating the clinical signs of DS. There is growing evidence indicates that PDT is highly efficacious in the management of various oral inflammatory diseases including oral lichen planus, oral mucositis, herpes labialis [17, 41, 42], which further substantiate the results of the present review.

It is pertaining to mention that the efficacy of PDT is governed by several important factors including type of the photosensitizers, source of light, oxygen availability, laser parameters, duration and frequency of the treatment [43]. Among these, the type of the photosensitizers is the most important factor that influences the therapeutic efficacy of PDT. Unfortunately, the included studies showed a wide heterogeneity in the type of the photosensitizers and other related parameters such as the concentration and irradiation times of the photosensitizers, which, in turn, may have influenced the treatment outcomes. Another key factor that has a great influence on PDT efficacy is the source of light and the related factors (wavelengths, power density, and energy density). Again, the included studies showed great variability in this respect. For example, some studies used LED while others used diode lasers. Similarly, the wavelengths of the used laser/LED varied greatly across the studies, ranging from 455 to 940 nm. Such a discrepancy in PDT parameters is an obvious limitation, making comparability between studies very difficult, and thus no firm conclusion can be drawn. Further, lack of standardized methodologies precludes investigators from creating a standard protocol for the management of oral fungal infections including DS.

Although the findings of the present systematic review support the efficacy of PDT in the management of DS, some methodological shortcomings must be considered. One important limitation is the small sample sizes and the low quality of some of the included studies, and thus no concrete evidence can be concluded. Another key limitation is the marked heterogeneity across the included studies with respect to type of comparison group (the type of topically applied antifungal, dose, frequency, and duration), severity of DS, age and gender of the participants, frequency and duration of PDT sessions, follow-up period, outcome measures, type of photosensitizers, and other PDT-related parameters. Specifically, the wide discrepancy in PDT parameters impedes generating a common protocol that can be considered as a standard for use of PDT in DS treatment. Finally, most of the included studies (five studies) were conducted in one country (Brazil), and thus the generalizability of the results is questionable. Hence, conducting large-scale multicenter clinical trials is warranted.

## Conclusion

In conclusion, the results of the present updated systematic review and meta-analysis reveal that PDT is as efficacious as topical antifungal in the management of denture stomatitis, suggesting that PDT can be used as an alternative or as an adjunct to the topical antifungal medications for the management of DS. Further well-designed randomized clinical trials with large sample sizes and standardized photodynamic therapy parameters are required to discern the efficacy of PDT in the management of DS.

### Supplementary Information

**Additional file 1.**

## Data Availability

All data generated or analyzed during this study are included in this published article.

## Abbreviations

PDT: Photodynamic therapy
DS: Denture Stomatitis
MD: Mean Difference
CI: Confidence Interval
OR: Odds Ratio
PICOS: Participants, Intervention, Comparison, Outcomes, and Study Design
RCT: Randomized Clinical Trials
