# Supplementary material for: Efficacy of photodynamic therapy on candida colonization and clinical symptoms in denture stomatitis: a systematic review and meta-analysis
Source: BMC Oral Health. 2024 Jan 16;24:84. doi: 10.1186/s12903-023-03789-z (PMC10790471; doi:10.1186/s12903-023-03789-z)
Supplement: Supplementary file 1 — Additional file 1. [file 12903_2023_3789_MOESM1_ESM.docx]

| **Supplementary Table 1. Databases: Applied search strategy, and numbers of retrieved studies**  . | | | |
| --- | --- | --- | --- |
| Databases | Search strategy used | | Hits |
| MEDLINE searched via PubMed searched on  June 08 , 2023  via www.ncbi .nlm.nih.gov/sites | | ("stomatitis, denture"[MeSH Terms] OR "denture stomatitis"[All Fields] OR "oral candidiasis"[All Fields]) AND ("Photochemotherapy"[MeSH Terms] OR "photodynamic therapy"[All Fields]) 73 | 73 |
| ISI web of science Core Collection was searched via web of knowledge on June 08, 2023  via apps.webofknowledge.com | Search # 1 ALL= ("denture stomatitis" OR "oral candidiasis" ) ALL fields 3310  Search #2: ("Photochemotherapy" OR "photodynamic therapy" ) (All Fields)  54,555  Search # 3: #1 AND #2 (ALL= ("denture stomatitis" OR "oral candidiasis" )) AND ALL=(( "Photochemotherapy" OR "photodynamic therapy" ))  88 | | 88 |
|  |  | |  |
| Scopus searched via Scopus on June 08, 2023 via https://www.scopus.com | TITLE-ABS-KEY ( ( "denture stomatitis" OR "oral candidiasis" ) AND ( "Photochemotherapy" OR "photodynamic therapy" ) ) 79 | | 79 |
| Google Scholar was searched via <https://scholar.google.com/> on June 05, 2022 and updated on June 08, 2023 | ("denture stomatitis" OR "oral candidiasis") AND ("Photochemotherapy" OR "photodynamic therapy") | | 200 |
|  | 2260 | |  |
| **Total** |  | |  |
| ProQuest search via:  https://www.proquest.com/ | ("denture stomatitis" OR "oral candidiasis") AND ("photodynamic therapy" OR photochemotherapy) 52 | | 52  492 |
| Total |  | |  |

**Supplementary Table 2: List of excluded studies and the reason for exclusion**

| # | **Author, year** | **Title** | **Reason for exclusion** |
| --- | --- | --- | --- |
| **1** | Mima et al, 2011 | Denture stomatitis treated with photodynamic therapy: five cases | Case report |
| **2** | Davoudi et al, 2018 | Role of laser or photodynamic therapy in treatment of denture stomatitis: systematic review | Systematic review |
| **3** | Grego et al, 2017 | Antimicrobial photodynamic therapy for infectious stomatitis in snakes: clinical views and microbiological findings | Not applied on humans |
| **4** | Oliveira et al, 2018 | Efficacy of antimicrobial photodynamic therapy in denture stomatitis: report of two cases | Case report |
| **5** | Alves et al, 2017 | Antimicrobial photodynamic therapy mediated by photodithazine in treatment of denture stomatitis: a case report | Case report |
| **6** | Fontes et al, 2017 | Op-candida-associated denture stomatitis treated with antimicrobial photodynamic therapy: four cases | Case report |
| **7** | Aly. 2012 | Effect of photodynamic therapy (laser) and sodium hypochlorite on candida albicans in complete denture: comparative study | Literature review |
| **8** | Senna. 2012 | Photodynamic antimicrobial therapy in treatment of denture stomatitis | Proceeding |
| **9** | Prazmo et al. 2017 | Photodynamic therapy in treatment of denture stomatitis | Literature review |
| **10** | Mima 2016 | Comments on “Methylene blue-mediated photodynamic inactivation followed by low-laser therapy versus miconazole gel in treatment of denture stomatitis” | Commentary |
| **11** | Abduljabbar et al. 2017 | Efficacy of photodynamic therapy in the inactivation of oral fungal colonization among cigarette smokers and non-smokers with denture stomatitis. | No control group |
| **12** | Bacali et al, 2021 | Association of Graphene Silver Polymethyl Methacrylate (PMMA) with Photodynamic Therapy for inactivation of halitosis responsible bacteria in denture wearers | Not related |
| **13** | Vale et al, 2019 | Evaluation of the treatment of halitosis with photodynamic therapy in older patients with complete denture: Protocol for a randomized, controlled trial | Not related |
| **14** | Alhenaki et al, 2021 | Disinfection of acrylic denture resin polymer with rose Bengal, methylene blue and porphyrin derivative in photodynamic therapy. | Not applied on humans |
| **15** | Fontes et al. 2020 | Is antimicrobial photodynamic therapy a treatment option for denture stomatitis? | Case report |
| **16** | Afroozi et al 2019 | Comparison of the efficacy of indocyanine green-mediated photodynamic therapy and nystatin therapy in treatment of denture stomatitis. | Intervention (PDT) combined with nystatin |
| **17** | Maciel CM el all. 2016 | Methylene Blue-Mediated Photodynamic Inactivation Followed by Low-Laser Therapy versus Miconazole Gel in the Treatment of Denture Stomatitis. | PDT was combined with laser therapy |
| **18** | Pereira et al 2015 | Pereira CA, Domingues N, Silva MP, Costa AC, Junqueira JC, Jorge AO. Photodynamic inactivation of virulence factors of Candida strains isolated from patients with denture stomatitis. J Photochem Photobiol B. 2015 Dec;153:82-9. doi: 10.1016/j.jphotobiol.2015.08.029. Epub 2015 Sep 1. PMID: 26398815. | In vitro |

1-Mima EGO, Pavarina AC, Sliva MM, Ribeiro DG, Vergani CE, Kurachi C, Bagnato VS. Denture stomatitis treated with photodynamic therapy: five cases. Oral Surg Oral Med Oral Pathol Oral Radiol Endod. 2011

2- Davoudi A, Ebadian B, Nosouhian S. Role of laser or photodynamic therapy in treatment of denture stomatitis: systematic review. J Prosthet Dent. 2018

3-Grego KF, Carvalho MPN, Cunha MPV, Knobl T, Pogliani FC, Dias JLC, Sant’Anna ST, Ribeiro MS, Sellera FP. Antimicrobial photodynamic therapy for infectious stomatitis in snakes: clinical views and microbiological findings. Photodiagnosis Photodyn Ther. 2017

4-Oliveira RBDS, Ramos PADS, Motovani PDP, Reys IG, Silva BAD, Tucci R, Fontes KBFDC. Efficacy of antimicrobial photodynamic therapy in denture stomatitis: report of two cases. Oral Surg Oral Med Oral Radiol Endod

5-Alves F, Alonso GC, Carmello JC, Mima EGO, Bagnato VS, Pavarina AC. Antimicrobial photodynamic therapy mediated by pjotodithazine in treatment of denture stomatitis: a case report. Photodiagnosis Photodyn Ther. 2017.

6- Fontes KBFC, Farias IBDS, Cappato LP, Silva BA, Azevedo RS, Tucci R, Junior AT. Op-candida-associated denture stomatitis treated with antimicrobial photodynamic therapy: four cases. Oral Surg Oral Med Oral Pathol Oral Radiol Endod. 2017.

7- Aly SME. Effect of photodynamic therapy (laser) and sodium hypochlorite on candida albicans in complete denture: comparative study. CU Theses. 2012.

8- Senna AM. Photodynamic antimicrobial therapy in treatment of denture stomatitis. Intr Nuclear Inform System. 2012

9- Prazmo EJ, Mielczarek AB. Photodynamic therapy in treatment of denture stomatitis. Protetyka stomatolo Prosthodon. 2017

10-Mima GO. Comment on “methylene blue-mediated photodynamic inactivation followed by low-laser therapy versus miconazole gel in the treatment of denture stomatitis. Repositorio Instituc UNESP.2016

11-Abduljabbar T, Al-Askar M, Baig MK, AlSowygh ZH, Kellesarian SV, Vohra F. Efficacy of photodynamic therapy in the inactivation of oral fungal colonization among cigarette smokers and non-smokers with denture stomatitis. Photodiagnosis Photodyn Ther. 2017.

12-Bacali C, Carpa R, Buduru S, Moldovan ML, Baldea I, Constantin A, Moldovan M, Prodan D, Rusu LMD, Lucaciu O, Catoi F, Constantiniuc M, Badea M. Association of Graphene Silver Polymethyl Methacrylate (PMMA) with Photodynamic Therapy for inactivation of halitosis responsible bacteria in denture wearers. Nanomaterials (Basel). 2021.

13-Vale KLD, Horliana ACRT, Romero SDS, Deana AM, Gonçalves MLL, Ferrari RAM, Bussadori SK, Fernandes KPS. Evaluation of the treatment of halitosis with photodynamic therapy in older patients with complete denture: Protocol for a randomized, controlled trial. Medicine (Baltimore). 2019

14-Alhenaki AM, Alqarawi FK, Alzahrani KM, Aldahiyan N, Naseem M, Vohra F, Abduljabbar T. Disinfection of acrylic denture resin polymer with rose Bengal, methylene blue and porphyrin derivative in photodynamic therapy. Photodiagnosis Photodyn Ther. 2021

15-Fantoes KBFC, Ramos PAS, Oliveira RBS, Barreto RL Tucci R, Silva BA, Barki MCLJM. Is antimicrobial photodynamic therapy a treatment option for denture stomatitis?. Oral Surg, Oral Med, Oral Patholo, Oral Radiolo. 2020

16-Afroozi B, Zomorodian K, Lavaee F, Zare Shahrabadi Z, Mardani M. Comparison of the efficacy of indocyanine green-mediated photodynamic therapy and nystatin therapy in treatment of denture stomatitis. Photodiagnosis Photodyn Ther. 2019

17-Maciel CM, Piva MR, Ribeiro MA, de Santana Santos T, Ribeiro CF, Martins-Filho PR. Methylene Blue-Mediated Photodynamic Inactivation Followed by Low-Laser Therapy versus Miconazole Gel in the Treatment of Denture Stomatitis. J Prosthodont. 2016

18. Pereira CA, Domingues N, Silva MP, Costa AC, Junqueira JC, Jorge AO. Photodynamic inactivation of virulence factors of Candida strains isolated from patients with denture stomatitis. J Photochem Photobiol B. 2015 Dec;153:82-9. doi: 10.1016/j.jphotobiol.2015.08.029. Epub 2015 Sep 1. PMID: 26398815.
